# The use of mifepristone in abortion associated with an increased risk of uterine leiomyomas

**DOI:** 10.1097/MD.0000000000006680

**Published:** 2017-04-28

**Authors:** Qi Shen, Li Shu, Hui Luo, Xiaoli Hu, Xueqiong Zhu

**Affiliations:** Department of Obstetrics and Gynecology, the Second Affiliated Hospital of Wenzhou Medical University, Wenzhou, Zhejiang, China.

**Keywords:** logistic regression analysis, mifepristone, risk factors, uterine leiomyomas

## Abstract

To investigate the association between widespread use of mifepristone in abortions and risk of uterine leiomyomas.

We conducted a case-control study of 305 patients with uterine leiomyomas between January 2011 and July 2012; 311 women with ordinary vaginitis were selected as controls during the same period. Data were collected by questionnaires (including past history, life history, menstruation history, reproductive history, abortion history, the use of mifepristone, and uterine leiomyomas risk factors) and calculated by univariate and multivariate conditional logistic regression analyses; odds ratios and its 95% confidence interval were calculated to estimate the risk for uterine leiomyomas.

Abortion with mifepristone was one of the risk factors for uterine leiomyomas, and the risk increased with increasing frequency of mifepristone use. Family history of uterine leiomyomas, body mass index, age at menarche, number of full-term delivery, and medical abortion history were also correlated with uterine leiomyomas.

The use of mifepristone in abortion will increase the risk to develop uterine leiomyomas.

## 1 Introduction

Uterine leiomyomas are among the most common tumors in women, affecting 20% to 50% of reproductive women, which can cause dysmenorrhea, abnormal uterine bleeding, menorrhagia,and infertility.^[1]^ Uterine leiomyomas are generally considered as hormonal-dependent tumors; progesterone and estrogen are essential for growth and maintenance of leiomyomas.^[2]^ Except invasive surgical procedure, medical therapies are more widely used for symptomatic patients, including gonadotropin-releasing hormone agonists (GnRHa) and progesterone modulators.^[3]^

Mifepristone (formerly known as RU-486), is an antiprogesterone drug that is effective in treatment of uterine leiomyomas, resulting in decreased leiomyomas size and relieved symptoms.^[4,5]^ Long-term administration of low-dose (5 or 10 mg daily for 1 year,^[6]^ 2.5 mg daily for 6 months,^[4]^ etc) mifepristone results in leiomyomas shrinkage and amelioration of symptoms. However, Eisinger et al^[6]^ reported that uterine leiomyoma re-growth occurs slowly after the cessation of mifepristone treatment, 5.7 months follow-up after cessation of mifepristone, almost 20% baseline of uterine volume increased compared with 12 months mifepristone treatment end. When GnRHa (another hormone replacement drug for uterine leiomyomas) treatment is stopped, uterine leiomyomas can also re-grow rapidly, and the uterine volume can reach or even exceed the baseline volume.^[7]^ It is a challenge for medical therapy of uterine leiomyomas.

Mifepristone has also been widely used for medical abortion, emergency contraception, softening the cervix before the surgical termination of pregnancy, and sensitizing the uterus to prostaglandins.^[8,9]^ The global abortion rate in 2008 was 28 per 1000 women aged 15 to 44 years.^[10]^ Medical abortion, because it is convenient to access and does not require surgical skills, could increase the ratios of medical abortion in abortion. In China, a large proportion (75%) of medical abortions occur in unmarried women or primiparas, and the rate of unmarried young women seeking repeated abortions is high.^[11,12]^ Currently, most hospitals in China use 150 mg (25 mg twice daily for 3 days; total 150 mg) of mifepristone as the standard for early medical abortions (gestational age within 7 weeks, but some hospitals’ treatment can last more than 7 weeks).^[13]^

Mifepristone in the treatment of uterine leiomyomas follows a long-term and low-dose drug administration, whereas mifepristone in multiple medical abortions is repeatedly used for a short time-course and at a high dose. Considering the phenomenon that leiomyomas re-grow after cessation of mifepristone,^[6]^ we wonder whether mifepristone repeated for short courses and at high doses may show similar effects as the long-term and low-dose protocol in tumor recurrence, which may induce tumorigenesis or promote growth of uterine leiomyomas. However, no evidence of this association has been reported to date.

Thus, we hypothesize that the widespread use of mifepristone in abortions probably increases the risk for uterine leiomyomas. We collected data from a large sample of Chinese women in a case-control study, and analyzed the factors associated with uterine leiomyomas by univariate and multivariate statistical approaches. Subsequently, we explored the relation between uterine leiomyomas and the use of mifepristone in abortion, which may have important implications for the appropriate administration of mifepristone in the future.

## 2 Materials and methods

### 2.1 Study population and questionnaire design

This retrospective questionnaire-based study was conducted at the Department of Obstetrics and Gynecology, the Second Affiliated Hospital of Wenzhou Medical University, between January 2011 and July 2012.

Data were collected by trained interviewers from cases and controls by interview, with a standardized pretested questionnaire. The study collected information as follows: patient's basic information (age, height, weight, educational background, family history of uterine leiomyomas), living habits (smoking history and drinking), related laboratory test results (blood routine test, liver and kidney function, plasma hormone levels, and other data), complication (diabetes mellitus, hypertension, infertility, pelvic inflammation), choice of abortion method (medical abortion, artificial abortion), choice of contraceptive method (oral contraceptives, intrauterine devices, condoms, tubal ligation, safe period contraception, and other methods), medical abortion (number of times performed, drug category including dosage and usage), artificial abortion (number, dosage and usage frequency of mifepristone before operation), uterine leiomyomas (time of diagnosis on B ultrasound, uterus size, number, location and size of leiomyomas), and follow-up data.

After questionnaire, 305 cases with uterine leiomyomas were selected by B-ultrasound or surgical pathology confirmed, 311 controls were selected from women who were visiting gynecologist for ordinary vaginitis during the same period and without uterine leiomyomas (after selected as controls, they had regular physical examination with B-ultrasound every year; if anyone were examined with uterine leiomyomas anytime, the patients were excluded out from the control group). In case group (the first confirmed diagnosis of uterine leiomyomas) and control group, premenopausal women, exclusion criteria were as follows: uterine adenomyosis, abnormal cardiovascular, liver, and kidney function, use of oral contraceptive, use estrogen and progesterone drugs, use mifepristone for the treatment of uterine leiomyomas, use any intrauterine device. The study protocol was approved by the local ethics committee and the study was conducted in accordance with declaration of Helsinki. Informed consent was obtained from all individual participants included in the study.

### 2.2 Dosage and usage of mifepristone

For patients within 7 weeks of gestation and gestational sac diameter ≤2 cm by B-ultrasound, mifepristone was taken 25 mg per time and twice daily (2 doses 12 hours apart orally for 3 days; total 150 mg); misoprostol was taken 600 mg orally on the fourth morning.

At a gestational age of 7 to 9 weeks, mifepristone was taken 25 mg twice on the first evening, 25 mg twice daily (2 doses 12 hours apart orally) for the second and third days, misoprostol 400 mg vaginally on the fourth day, and curettage was given after 0.5 to 1 hour of last drug. There were special doctors who did the operation in induced-abortion room.

### 2.3 Statistical analysis

All statistics were performed using SPSS for Windows version 18.0 (Chicago, IL). Continuous variables were compared with unpaired *t* test or Mann–Whitney *U* test, and categorical variables were analyzed by chi-square test. First, relationships between interest factors and uterine leiomyomas were assessed by the univariate analyses. Then the significant variables were included in the multivariate logistic regressions to calculate odds ratios (ORs) and 95% confidence intervals (CIs). All tests were 2-tailed, and *P* < .05 was considered to be statistically significant in all statistical analyses.

## 3 Results

### 3.1 The baseline characteristics of cases and controls

As shown in Table 1, the age of the cases was 43.5 ± 7.56 and that of controls was 42.9 ± 7.07 years. There were no statistically significant differences between cases and controls in terms of age and educational background. The main clinical manifestations of cases with uterine leiomyomas: menstrual changes of 98 cases (32.1%), abdominal pain in 21 cases (6.9%), abdominal mass in 15 cases (4.9%), 11 cases of irregular vaginal bleeding (3.6%), there was pressure effects in 17 cases (5.6%), 14 cases with secondary anemia (4.6%), 185 patients with no obvious symptoms (60.7%).

**Table 1:**
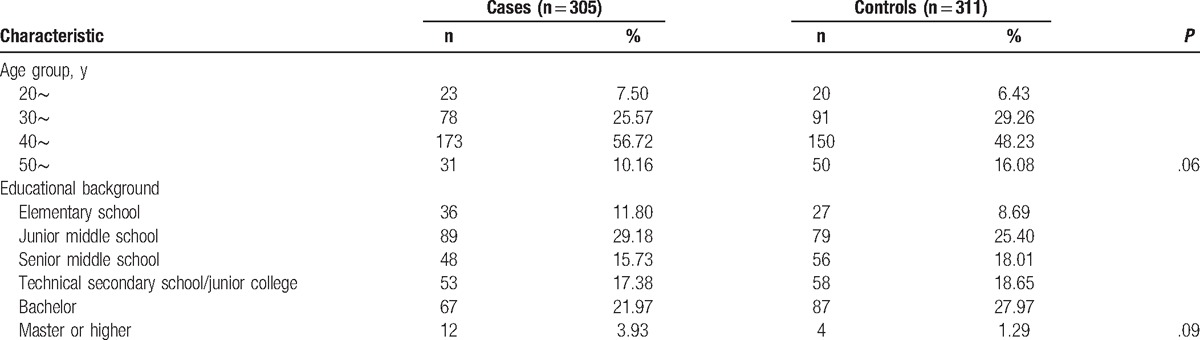
Age and educational background distribution of cases and controls.

### 3.2 The association between past history, life history, and uterine leiomyomas

As shown in Table 2, chi-square test showed that smoking history, passive smoking, drinking history, diabetes mellitus, hypertension history, infertility history, and pelvic inflammation were not obviously correlated with uterine leiomyomas. The occurrence of family history of uterine leiomyomas was positively correlated with uterine leiomyomas (OR 1.73, 95% CI 1.26–2.38, *P* < .01). Body mass index was also positively correlated with uterine leiomyomas (*P* < .01), body mass index ≥30 versus <18.5 (OR 2.06, 95% CI 1.17–3.61).

**Table 2:**
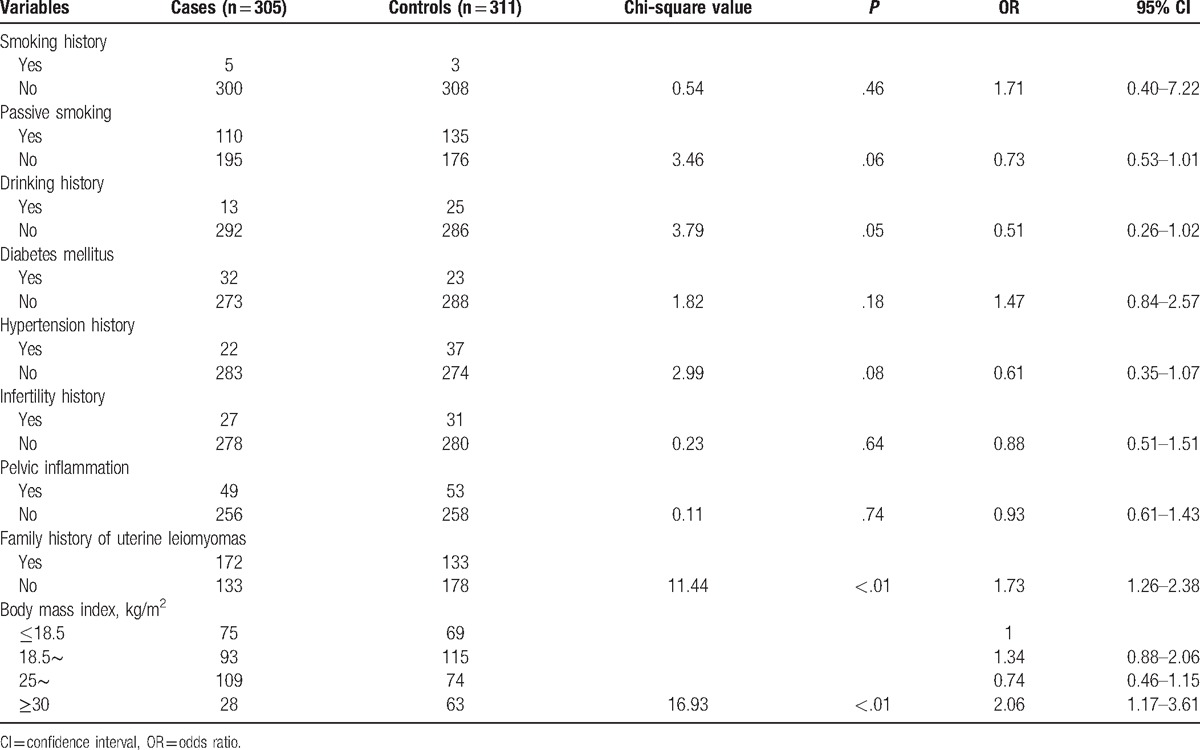
Association between past history/life history and uterine leiomyomas.

### 3.3 The association between menstruation history and uterine leiomyomas

As shown in Table 3, chi-square test showed that menstrual cycle, menstrual period, menstrual blood loss, and dysmenorrhea degree were not obviously correlated with uterine leiomyomas. Age at menarche was positively correlated with uterine leiomyomas (*P* < .01), and OR increased when the age at menarche lesser. For age at menarche <12, OR was 3.07 and 95% CI was 1.73 to 5.47.

**Table 3:**
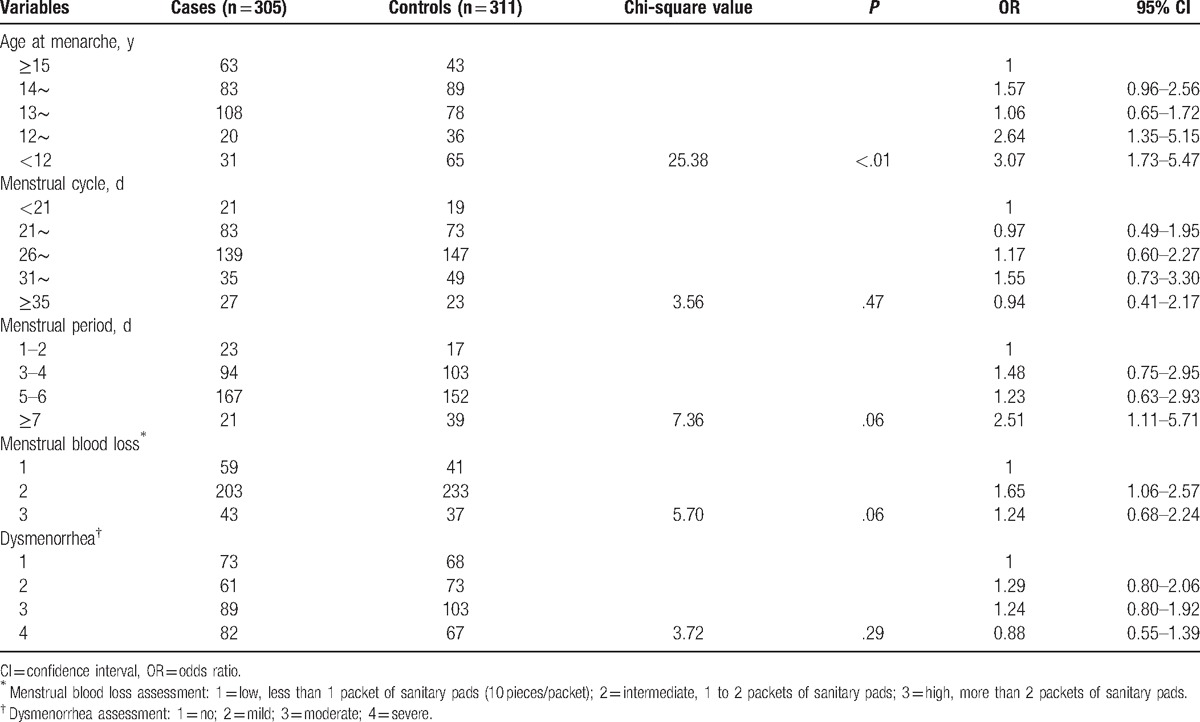
Association between menstruation history and uterine leiomyomas.

### 3.4 The association between reproductive history and uterine leiomyomas

As shown in Table 4, the risk of multipara women diagnosed uterine leiomyomas was 0.66 of nulliparous women (*P* = .04, 95% CI 0.44–0.99). The risk decreased when the number of full-term delivery increased (risk = 0.45 as the number ≥4 vs 1). Besides, the risk decreased when the age at last delivery increased (*P* = .04).

**Table 4:**
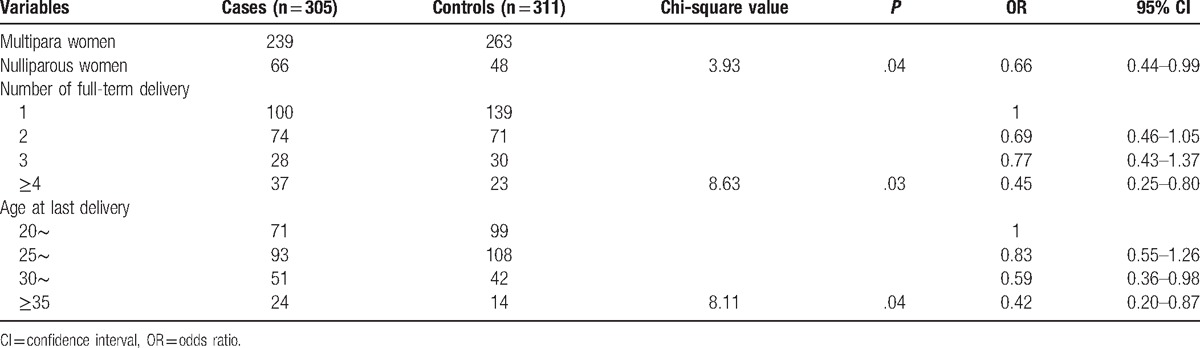
Association between reproductive history and uterine leiomyomas.

### 3.5 The association between abortion history and uterine leiomyomas

As shown in Table 5, artificial abortion history was positively correlated with uterine leiomyomas occurrence (*P* < .01, OR 1.64, 95% CI 1.17–2.30), the occurrence of mifepristone combined curettage with uterine leiomyomas also showed a positive correlation (*P* < .01, OR 1.95, 95% CI 1.29–2.94), but curettage alone was not an obvious risk factor (*P* = .07, OR 0.71, 95% CI 0.48–1.03). Mifepristone combined curettage ≥3 times (OR 2.67, 95% CI 1.22–5.85), the last time artificial abortion >5 years (OR 4.72, 95% CI 2.99–7.46), and uterine leiomyomas were positively related. Therefore, mifepristone use might be the main reason for artificial abortion history as 1 of uterine leiomyomas risk factors.

**Table 5:**
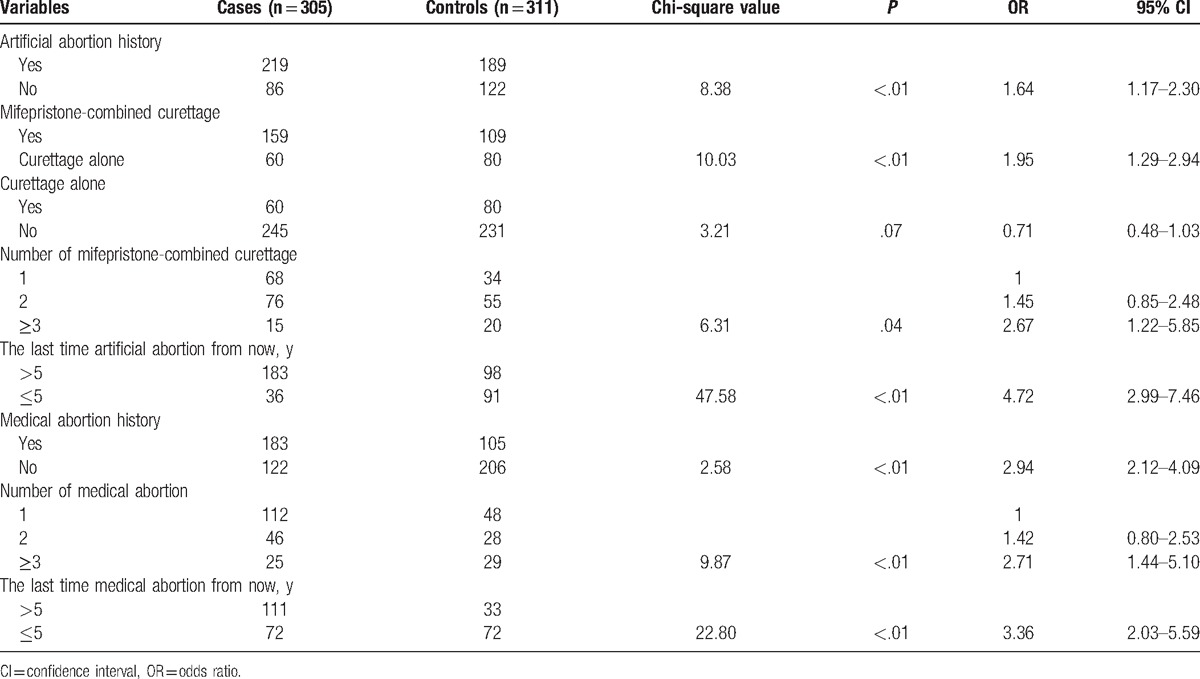
Association between abortion history and uterine leiomyomas.

Medical abortion and the incidence of uterine leiomyomas was positively correlated (*P* < .01). Both medical abortion ≥3 times (OR 2.71, 95% CI 1.44–5.10) and the last time medical abortion >5 years (OR 3.36, 95% CI 2.03–5.59) were positively correlated with uterine leiomyomas (*P* < .01). Mifepristone was one of the risk factors of uterine leiomyomas, and the risk increased when the frequency of use increased.

### 3.6 Multivariate logistic regressions

As shown in Table 6, all the significant variables were included in the multivariate logistic regressions, after analysis, and finally, the multifactor regression equation factors included were family history of uterine leiomyomas (X_1_), body mass index (X_2_), age at menarche (X_3_), mifepristone combined curettage (X_4_), number of full-term delivery (X_5_), and medical abortion history (X_6_). The regression equation was as follows: 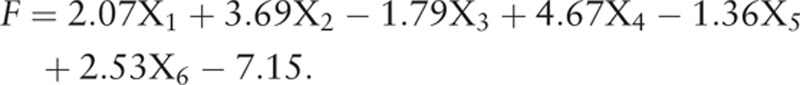


**Table 6:**
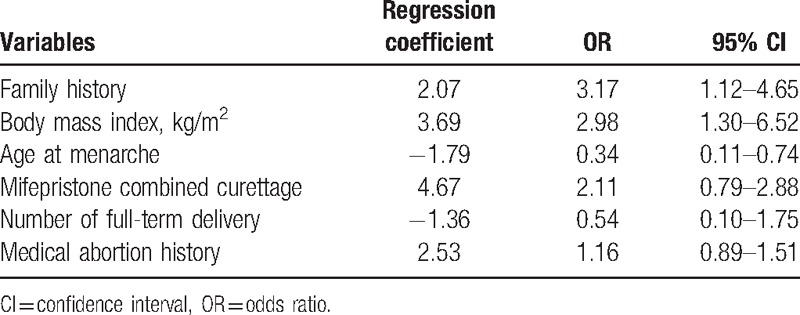
The result of multivariate logistic regressions.

## 4 Discussion

To our knowledge, this is the first questionnaire-based study to retrospectively assess the association between the use of mifepristone in abortion and uterine leiomyomas risk. There were also positive correlations between family history of uterine leiomyomas, body mass index, mifepristone combined curettage, medical abortion, and uterine leiomyomas risk. In contrast, age at menarche and full-term delivery were negatively associated with uterine leiomyomas risk.

Many evidences suggest that family history is a positive correlation factor for the development of uterine leiomyomas, which is due to genetic effects.^[14–16]^ Body mass index is also a known positive correlation factor. For instance, too much body obesity is related to decreased sex hormone-binding globulin levels, changed insulin receptors, and altered estrogen metabolism, which may promote the leiomyomas development.^[17–20]^

Age at menarche is a negative correlation factor with uterine laiomyomas, a common explanation as to why women with early age at menarche might have a hormonal environment different from that of women with later age at menarche. Furthermore, early age at menarche has been associated with increased levels of estradiol and estrone, but lower levels of sex hormone-binding globulin. Each year, the accumulated hormonal cycles may confer additional risk for developing leiomyomas.^[21–23]^ Number of full-term delivery is also a negative correlation factor. Moreover, women would not have menstruation during full-term pregnancy and total menstrual cycle numbers are relatively reduced. A full-term pregnancy may cause the expression of growth factors and ovarian hormones to drastically change, such as the levels of prolactin, urinary and plasma estradiol decrease, and the level of sex hormone-binding globulin increase. In addition, a full-term pregnancy may decrease the expression of estrogen receptor in uterine myometrium, resulting in decreased sensitivity of myometrial tissue to hormonal stimulation. In other words, the process of degradation and remodeling in uterine tissue could start up during and after full-term pregnancy period.^[24]^ Additionally, during the uterine recovery process, the nutrient blood vessels of original leiomyomas may be blocked, making leiomyomas gradually reduced in size.^[24–26]^ Our results are consistent with these studies.

Mifepristone can effectively reduce uterine and leiomyoma volumes and significantly alleviate leiomyoma symptoms (long-term and low-dose),^[5]^ but leiomyomas re-grow after cessation of mifepristone,^[6]^ and the exact mechanism remains unclear. Mifepristone has also been widely used for emergency contraception and medical abortion.^[27,28]^ Mifepristone treatment in medical abortion (short-course and high-dose) may show similar effects as uterine leiomyomas re-grow, which occurs due to drug cessation after long-term and low-dose drug use. In this study, we found that both the cases and the controls had high rates of artificial and medical abortions. The reason being that China started a family planning program in the 1970s and began implementing the 1-child-per-couple policy in 1979, aimed at curbing growth in the world's most populous nation.^[29]^ Mifepristone in abortion was 1 of the risk factors of uterine leiomyomas, and the risk increased when the frequency of use increased. The number of mifepristone-combined curettage or medical abortion significantly increased the risk of uterine leiomyomas.

The strength of our study is that mifepristone is 1 of the risk factors of uterine leiomyomas; when its use frequency increases, the risk increases. We recommend that more studies should be conducted to explain the mechanism of mifepristone use in abortion and uterine leiomyomas risk.

The major limitation of this study was its retrospective design, causing an inevitable recall bias. However, the large number of participants who completed the questionnaire strengthens the assessment of symptoms. Another problematic limitation of the study is that patients who fully knew they had uterine leiomyomas may be biased to report a higher impact of their symptoms based on prior discussions with their interviewers. Finally, the inclusion and analysis of solely Chinese women is a major limitation of the study in terms of applicability to a broader population such as white or black women.

A 3-year prospective clinical trial is currently in progress in our hospital, which includes leiomyoma-free women and women who have been diagnosed with uterine leiomyomas. We collect and record follow-up data, including the use of mifepristone-related drugs. We will publish the results when it is finished, which may have important implications for the appropriate use of mifepristone in the future.

## Acknowledgment

We thank Kyle Pressley at the University of Texas Health Science Center at San Antonio for editing the manuscript.
